# Sex-Related Differences in the Relationship between Sugar-Sweetened Beverage Consumption and Cardiorespiratory Fitness: Results from Chinese Cross-Sectional Study on Children

**DOI:** 10.3390/children9091411

**Published:** 2022-09-17

**Authors:** Dongjun Zhang, Junmin Yang, He Liu, Ruibao Cai

**Affiliations:** 1 School of Physical Education, Minnan Normal University, Zhangzhou 363000, China; 2 Sports Science Research Center, Minnan Normal University, Zhangzhou 363000, China; 3 Department of Physical Education, Xinjiang University Research, Urumqi 830000, China; 4 School of Physical Education, Chizhou University, Chizhou 247000, China; 5 Sports Health Promotion Center, Chizhou University, Chizhou 247000, China

**Keywords:** sugar-sweetened beverage consumption, cardiopulmonary fitness, relationship, children, China

## Abstract

Sugar-sweetened beverage (SSB) consumption continues to increase among children, with adverse health effects, and China is no exception. Our study investigates the association between SSB consumption and cardiopulmonary fitness. We used stratified whole group sampling to investigate and test SSB consumption and cardiopulmonary fitness in 21,055 children aged 13–15 years in China. A chi-square test and one-way ANOVA were used to compare different categories of SSB consumption. General linear regression analysis and logistic regression analysis were used to analyze the relationship between different SSB consumption and cardiopulmonary fitness in Chinese children. Our research results show the proportions of Chinese children with SSB consumption ≤ 1 time/week, 2–4 times/week, and ≥5 times/week were 33.3%, 52.8%, and 13.9%, respectively. VO_2max_ in children consuming ≥ 5 times/week was lower than those consuming 2–4 times/week and ≤2 times/week of SSB by 0.15 and 0.301 mL·kg^−1^·min^−1^, with statistically significant differences (*F*-value 18.807, *p* < 0.001). After relevant confounders were adjusted, children in the SSB consumption ≥ 5 times/week group had a higher risk of developing poorer cardiopulmonary fitness than those in the SSB consumption ≤ 1 time/week group (*OR*: 1.336, 95% *CI*: 1.181, 1.511) (*p* < 0.001). In conclusion, the consumption of SSBs among children aged 13–15 in China is higher than the recommended intake by the World Health Organization, and boys are higher than girls. In addition, after adjusting for relevant confounders, the association between SSB consumption and an increased risk of poor cardiorespiratory fitness remained. The relationship between SSB consumption and cardiopulmonary fitness was higher in girls compared with boys.

## 1. Introduction

Cardiorespiratory fitness (CRF), as an important component of physical fitness, is closely related to people’s health because of its association with cardiometabolic risk factors [1,2] and other health-related variables [3,4]. However, the CRF of children around the world has declined significantly over the past four decades [5], especially since the global pandemic of COVID-19 [6,7], which has led to the closure of schools and limited sports facilities [8]. In China, the CRF of children also decreased gradually from 1985 to 2010, and has been lower than that of Japanese children in recent years [9]. The persistent decline in CRF in Chinese children is influenced by a combination of factors, including lifestyle changes, declining physical activity levels, continued increases in academic stress, prolonged video screen behavior, and increased high-energy diets [9].

In recent years, sugar-sweetened beverage (SSB) consumption has become a public health issue among children [10]. Many studies have shown that high SSB consumption intake is a potential risk factor for mental illness [11] and cardiovascular and metabolic diseases [12,13]. Although SSB consumption has declined over the past 15 years, it remains high among children [14,15]. In China, more than half of children consume SSBs [16]. Studies have shown that increased intake of SSBs by children will lead to the development of obesity and have a negative impact on physical fitness [12].

Several studies have confirmed the relationship between increased SSB intake and cardiometabolic disease risk in young people [17,18,19,20]. It was reported that CRF was associated with better cardiometabolic health [20]. However, the relationship between SSB consumption and CRF remains unclear. Given the increased SSB consumption in China, the present study aimed to estimate the relationship between SSB consumption and CRF. Our study hypothesized that there was a relationship between SSB and CRF in Chinese children and that there was a negative association between the two.

## 2. Materials and Methods

### 2.1. Data Sources and Participants Recruitment

Our study subjects were drawn from the Fujian, Anhui, and Xinjiang regions of China in a cross-sectional survey study. Subjects were sampled in three steps. First, according to the distribution range of each province in China, Fujian, Anhui, and Xinjiang were selected as the provinces for investigation in this study. Second, five middle schools were randomly selected in each province, and teaching classes ranging from 3–5 classes per grade level were selected according to the size of the schools. Third, in principle, the eligible middle school students in the class were used as the subjects of this study.

**Figure 1:**
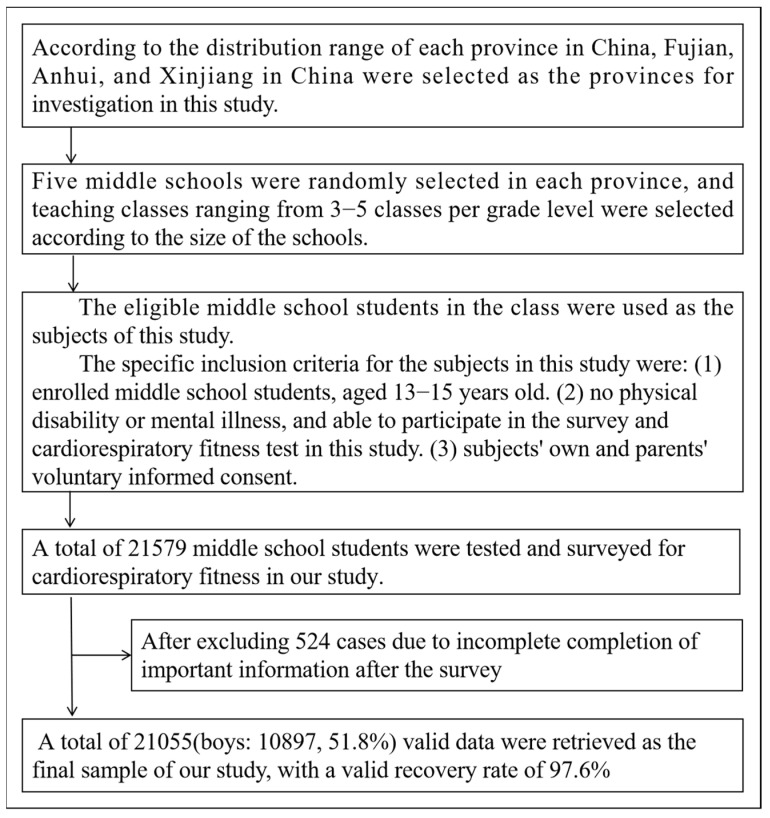
Flow chart of children aged 13–15 in China.

The inclusion criteria for the subjects in the present study were: (1) enrolled middle school students, aged 13–15 years old. (2) No physical disability or mental illness, and able to participate in the survey and CRF test in this study. (3) Subjects’ own and parents’ voluntary informed consent. According to the above sampling strategy, a total of 21,579 students were included in our study, and after excluding 524 cases due to incomplete compilation of important information, 21,055 valid data were retrieved as the final sample of our study, with a valid recovery rate of 97.6% for the questionnaire (Figure 1).

This study was carried out after being approved by the Ethics Committee of Chizhou University (NO: 20210506). Before the investigation, the written informed consent of the subjects and their parents was obtained.

### 2.2. Procedure

The staff in charge of the survey for this study were all trained and qualified master’s degree students. A uniform instructional language was used to explain the specific requirements of the survey to the subjects before the survey. The survey was administered electronically, and each participant scanned a QR code after class to conduct the survey. The content of the questionnaire consisted of basic demographic information, SSB consumption, and covariate questionnaires. To ensure the validity of the survey, the subjects’ test was filled out on site, and any questions were explained by the test staff.

### 2.3. Sugar-Sweetened Beverage Consumption

SSB consumption was conducted using a questionnaire from the China Student Survey on Physical Fitness and Health (CNSSCH) [21]. The validity and reliability of the questionnaire have been verified in previous studies [22,23]. SSB consumption investigated the number of times the subjects consumed sugary beverages in the past 7 days, calculated as 250 mL each time. SSBs included Coke, Sprite, fruit juice, Red Bull, etc. The frequency was 0–5 times/week, and our study was trichotomized into ≤1 time/week, 2–4 times/week, and ≥5 times/week to ensure that each group consisted of enough subjects.

### 2.4. Cardiorespiratory Fitness

CRF is tested indirectly using the 20-m shuttle run test (20-m SRT). The 20-m SRT was first proposed by Léger in 1984 and is now used in more than 50 countries worldwide to measure CRF in children [24]. CRF uses maximum oxygen uptake (VO_2max_) as an important evaluation index, and its reliability has been confirmed by relevant studies. The equation developed by Léger et al. focuses on deriving VO_2max_ levels in children as follows: VO_2max_ = 31.025 + 3.238 × S − 3.248 × age + 0.1536 × S × age. S denotes the last maximum velocity of the tester and S = 8 + 0.5 × the highest level reached by the tester.

### 2.5. Covariant

The covariates in our study included the father’s education, the mother’s education, screen time, duration of physical activity, body mass index, waist circumference, breakfast, and snacks. Father’s and mother’s education were categorized as elementary school and below, junior high school, high school, college, and above, and the duration of screen time was the average daily time spent watching TV, cell phone, tablet, etc. in the past 7 days. The duration of physical exercise was the average daily duration of exercise in the past 7 days, which was divided into <30 min/d, 30–60 min/d, and >60 min/d. The height and weight measurements were tested according to the testing methods and instruments required [25]. The body mass index was derived from the weight (kg)/height (m)^2^. Breakfast and snacks were the frequency of the subjects in the past 7 days, and our study was divided into ≤1 time/week, 2–3 times/week, and ≥4 times/week.

### 2.6. Statistical Analyses

Mean ± standard deviation (M ± SD) was used to present continuous variables and percentages for categorical variables. The difference in SSB consumption was made using one-way ANOVA (one-way analysis of variance). Comparisons of categorical variables between different SSB consumption were performed by chi-square test.

General linear regression (continuous variables) and logistic regression analysis (categorical variables) were used to analyze the relationship between SSB consumption and CRF among Chinese children, respectively. Three different models were used in the analysis: Crude Model did not adjust for any covariates; Model I adjusted for waist circumference, gender, father’s education, mother’s education, screen time, duration of physical activity, body mass index, and age. Model II adjusted for breakfast and snack factors based on model I. We set a dummy variable for SSB consumption and used it as a continuous variable to estimate the dose-response relationship between SSB consumption and CRF. The CRF differential was calculated after stratifying by age and gender VO_2max_ and standard deviation, and subjects were identified as poor CRF when their results were below the mean of 1SD.

The data of our study were analyzed using SPSS 25.0 (Armonk, NY, USA) and GraphPad Prism 8.0.2 software. Bilateral *p* < 0.05 was the level of statistical significance.

## 3. Results

Among 21,055 Chinese children aged 13–15 years, there were 10,897 boys (51.8%). The mean age of all subjects was 14.06 ± 0.82 years. The proportions of Chinese children SSB consumption ≤ 1 time/week, 2–4 times/week, and ≥5 times/week were 33.3%, 52.8%, and 13.9%, respectively. The consumption of SSBs was higher among boys (51.8%) than girls (48.2%).

In terms of different categories of father’s education, mother’s education, screen time, duration of physical activity, breakfast, and snacks, the proportions of SSB consumption were significantly different (χ^2^ = 69.666, 76.836, 492.666, 118.145, 324.607, 3114.256, *p <* 0.001). Among children with SSB consumption ≥ 5 times/week, boys, those with father’s education and mother’s education as high school, screen time < 60 min/d, and duration of physical activity < 30 min/d, breakfast ≥ 4 times/week, and snacks ≥ 4 times/week were the highest among children (Table 1).

One-way ANOVA showed that there were statistically significant differences in height, weight, waist circumference, 20-m SRT, and VO_2max_ among children with different SSB consumption (*F*-values were 59.342, 27.520, 132.088, 15.321, 18.807, respectively, *p* < 0.001) (Table 2).

VO_2max_ was statistically lower in Chinese children and adolescent boys and girls with SSB consumption ≥ 5 times/week than in those with 2–4 times/week and ≤1 time/week (*F*-value = 23.866, 42.172, *p* < 0.001). Overall, the VO_2max_ of children with SSB consumption ≥ 5 times/week was lower than those with 2–4 times/week and ≤1 time/week by 0.15 and 0.301 mL·kg^−1^·min^−1^, respectively (*F*-value = 18.807, *p* < 0.001) (Table 3).

As seen in Model II, adjusted for relevant confounders, compared to those with SSB consumption ≤ 1 time/week, Chinese children with SSB consumption ≥ 5 times/week had VO_2max_ decreased by 1.077 mL·kg^−1^·min^−1^ (*p <* 0.001) for boys and 1.058 mL·kg^−1^·min^−1^ (*p <* 0.001) for girls. Overall, after adjusting for relevant influences, VO_2max_ was reduced by 1.071 mL·kg^−1^·min^−1^ in those with ≥5 times/week compared with those with SSB consumption ≤ 1 time/week in Chinese children (*p* < 0.001). There is a dose–response relationship between SSB consumption and VO_2max_ (Table 4).

Overall, after adjusting for relevant influences (Model II), children in the SSB consumption ≥ 5 time/week group had a higher risk of poorer CRF than children in the SSB consumption ≤ 1 time/week group (*OR*: 1.336, 95% *CI*: 1.181, 1.511) (*p* < 0.001). The same trend (*p <* 0.001) was found in Chinese adolescent boys (*OR*: 1.147, 95% *CI*: 0.977, 1.346) and girls (*OR*: 1.713, 95% *CI*: 1.413, 2.077) (Table 5). Our findings suggest that, compared to boys, the CRF of girls is more susceptible to SSB consumption. Higher SSB consumption in Chinese children was associated with a higher risk of developing lower CRF, indicating lower VO_2max_ levels.

## 4. Discussion

Our study used cross-sectional data to analyze the relationship that exists between SSB consumption and CRF among Chinese children. We found that Chinese children’s SSB consumption is related to poorer performance of CRF. After adjusting for age, gender, father’s education, mother’s education, screen time, duration of physical activity, body mass index, waist circumference, breakfast, and snack factors, SSB consumption and CRF in Chinese children were also related. Our study also found that SSB consumption was associated with poorer CRF, and the association was higher in girls compared to boys.

Our findings provide an update on SSB consumption for Chinese children aged 13–15 years. The proportions of Chinese children with SSB consumption ≤ 1 time/week, 2–4 times/week, and ≥5 times/week were 33.3%, 52.8%, and 13.9%, respectively. The data suggest that at least 66.7% of Chinese children have SSBs ≥2 times/week. This is similar to the previous results of more than five levels of children’s SSB consumption in China [26]. This is higher than the data on SSB consumption among Australian children aged 2–18 years (46.7%) [27]. The results of the study showed that Chinese boys (51.8%) had higher SSBs beverage consumption than girls (48.2%), which is consistent with the results of previous studies [28]. Boys tended to associate the behavior of SSB consumption with being “popular”, “cool”, and “adventurous”, and have positive attitudes toward SSB consumption [29,30]. However, girls tended to associate the behavior of drinking healthy beverages with maintaining good body shape, so they were more likely to choose plain water and pure fruit juices over SSBs [31].

The 20-m SRT is currently the most widely used method for on-site assessment of CRF in various countries and is used in at least 50 countries around the world [24]. In this study, 20-m SRT was used to estimate the VO_2max_, and the results showed that the VO_2max_ of 13–15-year-old boys in China was about 43.24 mL/kg/min, and that of 13–15-year-old girls was about 40.17 mL·kg^−1^·min^−1^. Previous studies showed that VO_2max_ was about 45.59 mL·kg^−1^·min^−1^ for Chinese boys and for girls aged 13–15 years VO_2max_ was about 40.96 mL·kg^−1^·min^−1^ [9]. Therefore, the CRF of Chinese children aged 13–15 years was lower compared with the results of previous studies. This may be related to the increase in BMI, decrease in physical activity, and continuous increase in SSB consumption among Chinese children in recent years [32].

After adjusting for relevant influencing factors (Model II), our findings suggest that the higher the SSB consumption in Chinese children, the higher the risk of developing lower CRF, indicating lower VO_2max_ levels. This is the same conclusion as a European study in which CRF was negatively associated with SSB consumption in children [33]. Some studies have shown that economic development has a negative impact on cardiorespiratory health in children [34]. However, there is growing evidence of a strong associated between SSB consumption and cardiometabolic health in children [35]. SSB consumption may have a direct effect on human cardiometabolic health independent of weight [36]. Notably, as previous studies have shown, children with heavy SSB consumption are more likely to have screen time ≥2 h, reduced milk intake, high meat consumption, and high energy diets [37,38]. Furthermore, despite statistical adjustment for some confounding factors, residual confounding factors still exist in observational studies. Thus, in addition to the relationship with SSB consumption, the overall aggregation effect of an “unhealthy” diet and sedentary behavior was also responsible for the decrease in CRF reported in this study. The overall aggregation effect of an “unhealthy” diet and sedentary behavior is also an important cause of decreased CRF.

Our results also showed that the OR of SSB consumption and VO_2max_ was higher in girls compared with boys in Chinese children, indicating a stronger association. This also suggests that more attention should be paid to the control of SSB consumption in Chinese child-adolescent girls, due to the gender differences found in this study. On the one hand, there is a strong correlation between physical activity level, duration of participation in physical training, and CRF of children, and there is a positive correlation between them [39]. Boys had higher levels of high-intensity physical activity and physical exercise time compared to girls [40]. The CRF was more likely to be influenced by the level of intense physical activity, and duration of physical exercise in boys, and lower in girls. Therefore, CRF of girls is more likely to be affected by dietary habits, which leads to the higher OR value of girls in this study; that is, the higher correlation between SSB consumption and CRF. On the other hand, boys are also more likely to meet the moderate to vigorous physic alactivity(MVPA) recommended amount compared to Chinese children and adolescent girls [41]. Research shows unhealthy dietary patterns, including red and processed meat, SSBs and salty snacks, starchy foods and refined carbohydrates, are associated with higher BMI [37]. In addition to this, differences in muscle strength, eating behavior, and health perceptions brought about by gender differences are also important reasons for the differences in their results.

Our study has several strengths. On the one hand, the larger sample can represent the reality of Chinese children. On the other hand, the study included as many confounding factors affecting CRF as possible, such as demographic factors, anthropometric indicators, and dietary behaviors, to better analyze the associated between SSBs beverage consumption and CRF. However, our study also has several limitations. First, the evaluation of VO_2max_ in this study was obtained by indirect test of 20-m SRT. Although 20-m SRT is a traditional and simple test method, which can test a large number of samples at the same time, it is still a CRF estimation method, and there is a certain deviation from the actual CRF direct measurement results. Second, because the study used a cross-sectional analysis, only the relationships that exist between factors can be analyzed. The specific effect of SSB consumption on the increased risk of poor CRF is uncertain. In future studies, prospective cohort studies should be used to analyze the causal relationship between SSBs and CRF. Third, SSB consumption was investigated using a questionnaire. Recall bias is unavoidable due to the influence of recall ability between subjects. Therefore, the true picture of SSBs may be underestimated. Finally, this study only surveyed students aged 13–15 years, with a limited age range. Future research should also expand the age range of the survey, such as children aged 6–12 and 16–18.

## 5. Conclusions

Our study showed that SSBs were generally higher in the 13–15-year-old group of Chinese children, and were higher in boys than in girls. In addition, after adjusting for relevant confounders, SSBs remained associated with an increased risk of poor CRF. There was a higher association between SSBs and CRF in girls compared to boys in Chinese children. Our study provides a timely update and complements the current understanding of SSB consumption among Chinese children. From a public health perspective, SSBs should be reduced and various actions should be taken to improve CRF levels. For example, the government should control the advertising related to SSB, increase the tax of SSB, advocate to increase the duration of physical exercise, and improve the level of physical exercise. In school health education, students should be guided to reduce the consumption of SSBs, especially for boys. In summary, governmental policymakers, schools, and parents should be united to improve the health education of Chinese children, develop healthy eating behaviors and habits, and encourage children to drink calorie-free beverages such as water rather than SSBs. They should also strengthen the duration of physical exercise and physical activity levels, thus improving the CRF level of Chinese children.

## Figures and Tables

**Table 1:** Percentage of SSB consumption among children with different characteristics in China.

Characteristics	SSB Consumption			Total	*χ* ^2^	*p*-Value
	≤1 Time/Week	2–4 Time/Week	≥5 Times/Week			
N	7021 (33.3%)	11,114 (52.8%)	2920 (13.9%)	21,055	7174.957	<0.001
Sex						
Boys	3251 (46.3%)	5898 (53.1%)	1748 (59.9%)	10,897 (51.8%)	168.107	<0.001
Girls	3770 (53.7%)	5216 (46.9%)	1172 (40.1%)	10,158 (48.2%)		
Father’s education						
Elementary school and below	786 (11.2%)	1206 (10.9%)	281 (9.6%)	2273 (10.8%)	69.666	<0.001
Junior high school	2510 (35.7%)	4170 (37.5%)	913 (31.3%)	7593 (36.1%)		
High school	2303 (32.8%)	3774 (34.0%)	1104 (37.8%)	7181 (34.1%)		
College and above	1422 (20.3%)	1964 (17.7%)	622 (21.3%)	4008 (19.0%)		
Mother’s education						
Elementary school and below	1145 (16.3%)	1833 (16.5%)	407 (13.9%)	3385 (16.1%)	76.836	<0.001
Junior high school	2454 (35.0%)	4006 (36.0%)	922 (31.6%)	7382 (35.1%)		
High school	2242 (31.9%)	3680 (33.1%)	1011 (34.6%)	6933 (32.9%)		
College and above	1180 (16.8%)	1595 (14.4%)	580 (19.9%)	3355 (15.9%)		
Screen time						
<60 min/d	4353 (62.0%)	5471 (49.2%)	1273 (43.6%)	11,097 (52.7%)	492.666	<0.001
60–120 min/d	1747 (24.9%)	3583 (32.2%)	859 (29.4%)	6189 (29.4%)		
>120 min/d	921 (13.1%)	2060 (18.5%)	788 (27.0%)	3769 (17.9%)		
Duration of physical activity						
<30 min/d	3340 (47.6%)	4612 (41.5%)	1302 (44.6%)	9254 (44.0%)	118.145	<0.001
30–60 min/d	2687 (38.3%)	4889 (44.0%)	1072 (36.7%)	8648 (41.1%)		
>60 min/d	994 (14.2%)	1613 (14.5%)	546 (18.7%)	3153 (15.0%)		
Breakfast						
≤1 time/week	232 (3.3%)	431 (3.9%)	248 (8.5%)	911 (4.3%)	324.607	<0.001
2–3 times/week	693 (9.9%)	1674 (15.1%)	537 (18.4%)	2904 (13.8%)		
≥4 times/week	6096 (86.8%)	9009 (81.1%)	2135 (73.1%)	17,240 (81.9%)		
Snacks						
≤1 time/week	2196 (31.3%)	1227 (11.0%)	196 (6.7%)	3619 (17.2%)	3114.256	<0.001
2–3 times/week	3708 (52.8%)	7268 (65.4%)	1027 (35.2%)	12,003 (57.0%)		
≥4 times/week	1117 (15.9%)	2619 (23.6%)	1697 (58.1%)	5433 (25.8%)		

Note: Each calculation of SSB consumption is calculated with a 250 mL bottle.

**Table 2:** One-way analysis of variance on health indicators of children with different SSB consumption in China.

Characteristics	SSB Consumption			Total	*F*-Value	*p*-Value
	≤1 Time/Week	2–4 Time/Week	≥5 Times/Week			
Age(years)	14.06 ± 0.82	14.05 ± 0.82	14.10 ± 0.81	14.06 ± 0.82	5.035	0.007
Height(cm)	164.38 ± 8.46	164.95 ± 8.62	166.44 ± 8.61	164.97 ± 8.59	59.342	<0.001
Weight(kg)	53.65 ± 10.72	54.08 ± 11.01	55.43 ± 11.32	54.12 ± 10.97	27.520	<0.001
Body Mass Index(kg/m^2^)	19.77 ± 3.20	19.78 ± 3.25	19.93 ± 3.36	19.80 ± 3.25	2.941	0.053
Waist Circumference(cm)	67.31 ± 8.31	68.43 ± 9.71	70.63 ± 9.91	68.36 ± 9.36	132.088	<0.001
20-m SRT(laps)	37.8 ± 16.19	37.16 ± 15.71	35.89 ± 14.79	37.2 ± 15.76	15.321	<0.001
VO_2max_·(mL·kg^−1^·min^−1^)	42.06 ± 4.77	41.92 ± 4.63	41.44 ± 4.47	41.9 ± 4.66	18.807	<0.001

Note: Abbreviations: 20-m SRT, 20-m shuttle run test. VO_2max_, maximal oxygen uptake. Each calculation of SSB consumption is calculated with a 250 mL bottle.

**Table 3:** VO_2max_ status of children in China with different consumption of SSBs.

SSB Consumption	N	VO_2max_ (mL·kg^−1^·min^−1^)		*F*-Value	*p*-Value
		Mean	Standard Deviation		
Boys					
≤1 time/week	3250	43.60	4.87	23.866	<0.001
2–4 times/week	5895	43.45	4.69		
≥5 times/week	1748	42.68	4.37		
Girls					
≤1 time/week	3762	40.74	4.25	42.172	<0.001
2–4 times/week	5206	40.18	3.90		
≥5 times/week	1172	39.59	3.96		
Total					
≤1 time/week	7012	42.06	4.77	18.807	<0.001
2–4 times/week	11,101	41.92	4.63		
≥5 times/week	2920	41.44	4.47		

Note: Abbreviations: N, sample size; VO_2max_, maximal oxygen uptake.

**Table 4:** Multiple linear regression analysis of VO_2max_ in children with different SSB consumption in China (n = 21,055).

VO_2max_	Estimates (95% CI)		
	Crude Model	Model I	Model II
Boys			
≤1 time/week	0.000 (Reference)	0.000 (Reference)	0.000 (Reference)
2–4 time/week	−0.149 (−0.350,0.052)	−0.138 (−0.337,0.061)	−0.256 (−0.458,−0.054)
≥5 times/week	−0.924 (−1.196,−0.651) ^a^	−0.838 (−1.110,−0.566) ^a^	−1.077 (−1.365,−0.789) ^a^
*p* for trend	<0.001	<0.001	<0.001
Girls			
≤1 time/week	0.000 (Reference)	0.000 (Reference)	0.000 (Reference)
2–4 time/week	−0.554 (−0.724,−0.385) ^a^	−0.559 (−0.719,−0.400) ^a^	−0.551 (−0.715,−0.388) ^a^
≥5 times/week	−1.148 (−1.413,−0.883) ^a^	−1.079 (−1.33,−0.828) ^a^	−1.058 (−1.325,−0.791) ^a^
*p* for trend	<0.001	<0.001	<0.001
Total			
≤1 time/week	0.000 (Reference)	0.000 (Reference)	0.000 (Reference)
2–4 time/week	−0.145 (−0.285,−0.006)	−0.354 (−0.482,−0.225) ^a^	−0.407 (−0.538,−0.276) ^a^
≥5 times/week	−0.626 (−0.827,−0.425) ^a^	−0.964 (−1.151,−0.777) ^a^	−1.071 (−1.270,−0.873) ^a^
*p* for trend	<0.001	<0.001	<0.001

Note: Abbreviations: CI, confidence interval. VO_2max_, maximal oxygen uptake. The Crude Model was not adjusted for any additional variables. Model I adjusted for age, sex, father’s education, mother’s education, screen time, duration of physical activity, body mass index, and waist circumference. Model II adjusted for breakfast, snack, and other factors based on Model I. ^a^ indicates *p* < 0.001.

**Table 5:** Logistic regression analysis of VO_2max_ in children with different SSB consumption in China (n = 21,055).

VO_2max_	Odds Ratio (95% *CI*)		
	Crude Model	Model I	Model II
Boys			
≤1 time/week	1.000	1.000	1.000
2–4 time/week	0.935 (0.837,1.046)	0.924 (0.825,1.036)	0.945 (0.842,1.062)
≥5 times/week	1.175 (1.016,1.359)	1.113 (0.958,1.293)	1.147 (0.977,1.346)
*p* for trend	<0.001	<0.001	<0.001
Girls			
≤1 time/week	1.000	1.000	1.000
2–4 time/week	1.157 (1.023,1.309)	1.146 (1.009,1.302)	1.131 (0.992,1.289)
≥5 times/week	1.769 (1.491,2.100) ^a^	1.767 (1.475,2.117) ^a^	1.713 (1.413,2.077) ^a^
*p* for trend	<0.001	<0.001	<0.001
Total			
≤1 time/week	1.000	1.000	1.000
2–4 time/week	1.052 (0.969,1.142)	1.023 (0.940,1.113)	1.027 (0.942,1.120)
≥5 times/week	1.445 (1.294,1.614) ^a^	1.337 (1.192,1.501) ^a^	1.336 (1.181,1.511) ^a^
*p* for trend	<0.001	<0.001	<0.001

Note: Abbreviations: CI, confidence interval. VO_2max_, maximal oxygen uptake. The Crude Model was not adjusted for any additional variables. Model I adjusted for age, sex, father’s education, mother’s education, screen time, duration of physical activity, body mass index, and waist circumference. Model II adjusted for breakfast, snack, and other factors based on model I. ^a^ indicates *p* < 0.001.

## Data Availability

To protect the children’s privacy, the questionnaire data will not be disclosed to the public. They are available, upon request, from the authors of this article.
